# Self‐Reported Dysmenorrhea Among Adolescent Girls in the Cape Coast Metropolis: A Cross‐Sectional Study

**DOI:** 10.1002/hsr2.72008

**Published:** 2026-03-08

**Authors:** Amina S. Abugri, Attoh Tetteh, Gabriel P. Kotam, Angele Comlan‐Cataria, Stephen Ocansey, Richard K. D. Ephraim

**Affiliations:** ^1^ Department of Physician Assistant Studies School of Allied Health Sciences, University of Cape Coast Cape Coast Ghana; ^2^ Department of Medical Laboratory Science School of Allied Health Sciences, University of Cape Coast Cape Coast Ghana; ^3^ Department of Optometry and Vision Science School of Allied Health Sciences, University of Cape Coast Cape Coast Ghana

## Abstract

**Background and Aims:**

Dysmenorrhea is a common gynaecological condition among female adolescents and affects students' well‐being in Ghana. This study assessed dysmenorrhea prevalence, effects, and coping mechanisms among adolescent girls in Junior High Schools in the Cape Coast metropolis.

**Methodology:**

A cross‐sectional study was conducted among adolescent girls in three Junior High Schools in the Cape Coast Metropolis. Interviewer‐administered questionnaires were employed to obtain data from 198 postmenarchal adolescents aged 10–19 years. The data were analysed via STATA 15.1. Bivariate and multivariate logistic regression were used to establish the strength of associations between all significant variables, and the results are reported as crude and adjusted odds ratios.

**Results:**

Most adolescents (75.6%, *n* = 149) experienced menarche between the ages of 12 and 13 years; the least common age at menarche was 9. The prevalence of self‐reported dysmenorrhea was 86.9% (*n* = 172). Dysmenorrhea restricted adolescents from engaging in their normal physical activities (44.4%, *n* = 88) and led to poor concentration (39.4%, *n* = 78), social withdrawal (41.9%, *n* = 83), unnecessary irritation (42.9%, *n* = 85), decreased academic performance (15.2%, *n* = 30), and absenteeism (11.6%, *n* = 23). Adolescents who experienced early menarche had significantly lower odds of reporting severe dysmenorrhea compared to those with late menarche (AOR = 0.51, *p* = 0.02). Utilization of over‐the‐counter medications (26.8%, *n* = 53), ignoring pain (28.3%, *n* = 56), and resting (46.5%, *n* = 92) were the common coping mechanisms employed by adolescents.

**Conclusion:**

Dysmenorrhea is very prevalent among adolescents in the Cape Coast municipality and adversely affects their social and academic lives. This highlights a significant gap in healthcare access and education, pointing to the need for better public health strategies.

## 1 Introduction

Dysmenorrhea refers to painful uterine cramps that occur before or during menstruation [1]. The pain is usually localized in the lower abdomen and can radiate to the back and thighs [2]. Menstruation is associated with increased production of prostaglandins and vasopressin. While prostaglandins cause uterine contractions and pain, vasopressin release enhances uterine contractility and causes ischemic pain by inducing vasoconstriction [3]. Additionally, hormonal fluctuations during the menstrual cycle can cause dysmenorrhea [4]. Stress, diet, menstrual irregularities, menarchal age, and family relationships may influence menstrual pain intensity [5].

Dysmenorrhea is a prevalent gynaecological condition affecting a significant number of adolescent girls during their menstrual cycles. Studies have reported that the prevalence of dysmenorrhea is 60%–93% among adolescents [6]. In a cross‐sectional study among adolescents in the Greater Accra region of Ghana, Acheampong et al. (2019) reported that the prevalence of dysmenorrhea was 68.1% [3]. In a similar study in the northern region of Ghana, a prevalence of 80.9% was reported [7]. These high prevalence rates highlight the widespread nature of dysmenorrhea and raise concerns about its impact on adolescents' health and daily activities.

Dysmenorrhea adversely affects adolescents [8], as usual activities such as sitting, walking, and having a full bladder can cause or intensify menstrual pain [9]. Dysmenorrhea is the primary cause of recurrent short‐term school absenteeism among menstruating adolescents [10, 11]. It affects academic performance through interference with concentration and performance [12, 13]. Pain and discomfort can lead to emotional distress, increased irritability, and decreased participation in social and extracurricular activities [14, 15]. To annul this pain and discomfort associated with dysmenorrhea, adolescents rely on a variety of coping mechanisms, such as ignoring pain [3], heat therapy [16], rest [11], self‐medication [17], and a few consult physicians [3].

Despite its high prevalence and considerable impact on daily activities, dysmenorrhea is often poorly managed and frequently overlooked. Many young females endure pain in silence because of a lack of awareness, social stigma, and the normalization of menstrual discomfort. We used a cross‐sectional study design to collect data on the prevalence, effects, and coping mechanisms of dysmenorrhea among adolescents in the Cape Coast metropolis to provide insights into the burden of dysmenorrhea and to inform preventive and control strategies in the district. To the best of our knowledge, this is the first study among the adolescent population in the Cape Coast Municipality.

## 2 Methodology

### 2.1 Study Design, Duration, Site, and Sample Size

This cross‐sectional study was conducted among female adolescents in Junior High Schools (JHSs) in the Cape Coast Municipality. We collected data on the prevalence, effects, and coping mechanisms of dysmenorrhea from 11th July to 14th July 2023. We sent a cover letter and proposal to 10 basic and JHSs in the Cape Coast metropolis and received a positive response from three of the schools. Schools were selected based on the criterion that they had both JHS and primary, as the JHS level typically enrolls girls aged 10–19 years, which aligned with our target audience. Data were collected from Kwaprow M/A JHS (Kwaprow), Imam Khomeini Islamic JHS (Amamoma), and St. Anthony Anglican JHS (Akotokyir). These schools were public JHSs serving broadly similar catchment populations. No formal school‐level or institutional characteristics were collected. An initial form was distributed to participants to collect data on their age, previous intrauterine device use, history of pelvic infections or previous pelvic surgery or trauma and attainment of menarche to assess participants' eligibility. We collected data from 198 postmenarchal adolescent girls between the ages of 10 and 19. Participants were recruited based on their menstrual status, age, and the absence of secondary pelvic conditions.

### 2.2 Eligibility Criteria

JHS adolescent girls aged 10–19 years who had their first menstrual period were recruited for the study. Adolescents who had not reached menarche; those with mental or physical conditions that could affect their ability to participate in the study; those with pelvic pathologies such as sexually transmitted diseases, endometriosis, and urinary tract infections, which were identified through medical history collection prior to data collection; and those on antidepressants, contraceptives and students who could neither understand English nor Fante, were excluded from the study. The assessment conducted before recruitment revealed that none of the participants used hormonal contraception, and two were excluded because they had a history of pelvic infections.

#### 2.2.1 Data Collection

Pretested, interviewer‐administered paper questionnaires were used to collect data on dysmenorrhea. The questionnaire was divided into four sections: sociodemographics (age, gender, religious affiliation, guardian, relationship with guardian, guardian's educational level), obstetric and gynaecological characteristics, prevalence and impact of dysmenorrhea, and coping mechanisms. The questionnaire was semi‐structured with a combination of closed‐ and open‐ended questions. The questionnaire design aligned with the specific objectives of the study and the population's demographics after a thorough appraisal of the literature [9, 12, 17, 18, 19]. We defined a regular menstrual cycle as a constant 26–32‐day interval during each menstrual cycle [20]. We assessed the severity of dysmenorrhea via the visual analogue scale (VAS). The VAS assesses the female perception of pain and ranges from 0 to 10: no pain to unbearable. The scale was interpreted as mild pain (1–3), moderate pain (4–7), or severe pain (8–10) [7, 21]. Following pretesting and literature‐informed design, no responses were received outside the predefined categories. Accordingly, all responses were coded into the existing categories and analysed quantitatively. Responses were analysed quantitatively, with responses categorized and presented as frequencies and percentages. The questionnaire was administered on paper.

### 2.3 Data Analysis

The data were initially entered into Microsoft Office Excel (2016), organized and imported into STATA 15.1 (StataCorp LLC, Texas 77845, USA) for analysis. The respondents' sociodemographic, obstetric, and gynaecological data are presented in tables and figures. Continuous data are presented as the means with standard deviations, whereas categorical data are presented as frequencies and percentages. Associations were examined both for the overall prevalence of dysmenorrhea and separately for severe dysmenorrhea. Chi‐square (*χ* ^2^) analysis was used to explore any associations between severe dysmenorrhea and the sociodemographic, obstetric, and gynaecological characteristics of the adolescents. For all significant variables, bivariate and multivariate logistic regression were used to establish the strength of the associations, and the results are reported as adjusted odds ratios. All analyses were two‐sided, pre‐specified, and performed at a 95% confidence interval, and a *p*‐value < 0.05 was considered to indicate statistical significance.

## 3 Results

Among the 198 participants involved in this study, the majority were in their second years (40.4%) of JHS, were Christian (86.9%), and were between the ages of 14 and 16 years (70.2%) (Table 1). Only 52.0% (*n* = 103) of the participants lived with both parents, and 25.8% (*n* = 51) lived with only their mothers. The majority of the guardians of these adolescents were self‐employed (60.1%, *n* = 119) and had no formal education (68.7%, *n* = 136). We found that most adolescents (75.6%, *n* = 149) had their first menstrual period between the ages of 12 and 13 years; the least common age at menarche was 9 years, and the maximum was 17 years (Table 2). Only 8.6% (*n* = 17) of the girls could confirm that they had a regular menstrual cycle, with almost half of the participants (*n* = 98) without a regular cycle, and 41.9% were unsure if they had regular cycles or not. During their menstrual period, 58.6% (*n* = 116) of the participants experienced blood flow within 3–5 days, while 37.9% (*n* = 75) had menses lasting more than 5 days. The reported prevalence of female circumcision was 3.0% (*n* = 6) among girls. A prior history of abortion was reported by 1.5% (*n* = 3) of the participants.

**Table 1:** Association between dysmenorrhea and the sociodemographic characteristics of adolescent junior high school females.

Variable	Frequency (*n*)	Percentage (%)
Age category (years) [mean ± SD = 14.45 ± 1.22]		
12–13 years	47	23.74
14–16 years	139	70.20
17–18 years	12	6.06
Level/form		
One	64	32.32
Two	80	40.40
Three	54	27.27
Person reside with		
Guardian	35	17.68
Parent	163	82.32
Relationship with person residing with		
Mother	51	25.76
Father	8	4.04
Both mother and father	103	52.02
Auntie/uncle	17	8.59
Grandparents	16	8.08
Sister/step mother	3	1.52
Occupation of guardian		
Government employed	70	35.35
Self‐employed	119	60.10
Unemployed	9	4.55
Level of education of guardian		
No formal education	24	12.12
Formal education	136	68.69
Informal education	35	17.68
Others	3	1.52
Religion		
Christianity	172	86.87
Islam	26	13.13
Ethnicity		
Ashanti	35	17.68
Fante	117	59.09
Northerner	13	6.57
Ga/Ewe/Akuapem/Krobo	11	5.56
Mole Dagbani	18	9.09
Foreigner	4	2.02
Junior High School (JHS)		
I. K. Islamic JHS (Amamoma)	36	18.18
St. Anthony Anglican JHS (Akotokyir)	64	32.32
Kwaprow M/A JHS (Kwaprow)	98	49.49

*Note:* The data are expressed as the means ± SDs or *n* (%).

**Table 2:** Obstetric and gynaecological characteristics of junior high school adolescent females.

Variable	Frequency (*n*)	Percentage (%)
Age or menarche category (years) [mean ± SD = 12.47 ± 1.04]		
9–11 years	23	11.68
12–13 years	149	75.63
14–17 years	25	12.69
Adolescent has regular cycle		
No	98	49.49
Yes	17	8.59
Not sure	83	41.92
Number of days of regular cycle (*n* = 17)		
20–25 days	5	29.41
26–28 days	8	47.06
29–30 days	4	23.53
Duration of blood flow		
2 days or less	7	3.54
3–5 days	116	58.59
Greater than 5 days	75	37.88
Number of pads usually used		
Two or less	96	48.48
Three to four	88	44.44
Five or more	14	7.07
Adolescent female circumcised		
No	192	96.97
Yes	6	3.03
Having history of abortion		
No	195	98.48
Yes	3	1.52

*Note:* The data are expressed as the means ± SDs or *n* (%).

**Figure 1:**
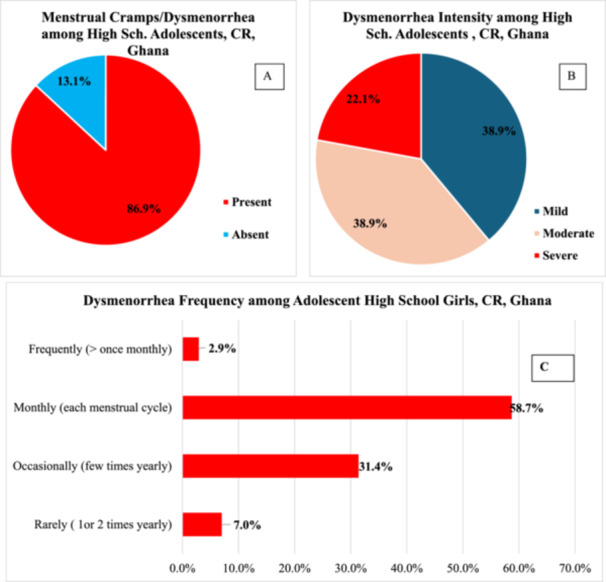
Graphs showing the prevalence (A), intensity (B), and frequency (C) of dysmenorrhea among high school adolescents in the Central Region (CR), Ghana.

The prevalence of dysmenorrhea was 86.9% (*n* = 172) (Figure 1A). While severe dysmenorrhea was reported by 38 adolescents (22.1%), the rest (77.9%) had either mild (*n* = 67) or moderate (*n* = 67) pain during their period of menstruation (Figure 1B). Approximately 26% (*n* = 45) had pain that lasted 3 days or more. The incidence of dysmenorrhea occurs monthly for most women (58.7%, *n* = 101), with 31.4% (*n* = 54) experiencing pain a few times a year (Figure 1C).

No significant associations were found between age category (*p* = 0.2), year of study (*p* = 0.08), relationship with guardian (*p* = 0.5), and occupation of guardians (*p* = 0.07). Age was not significantly associated with the overall prevalence of dysmenorrhea (Table 3); however, increasing age was associated with higher odds of severe dysmenorrhea (Table 4). A significant association was observed between school attendance and dysmenorrhea (*χ* ^2^ = 11.84, *p* = 0.004) (Table 3). We found that severe dysmenorrhea was significantly related to different school environments (*χ* ^2^ = 9.04, *p* = 0.02), and adolescent relationship with guardian (*χ* ^2^ = 12.40, *p *= 0.02) (Table 4). There was a significant difference in the ages of those who reported having severe dysmenorrhea (15.1 ± 0.2 years) compared with those who reported otherwise (14.3 ± 0.1 years) (*p* < 001) (Table 4).

**Table 3:** Association between dysmenorrhea and the sociodemographic characteristics of adolescent high school females.

Variable	Overall [*n* (%)]	Dysmenorrhea		*p*‐value**
		No [*n* (%)]	Yes [*n* (%)]	
Age category (years) [mean ± SD = 14.45 ± 1.22]				0.190
12–13 years	47 (23.74)	9 (34.62)	38 (22.09)	
14–16 years	139 (70.20)	17 (65.38)	122 (70.93)	
17–18 years	12 (6.06)	0 (0.00)	12 (6.98)	
Level/form				0.077*
One	64 (32.32)	13 (50.00)	51 (29.65)	
Two	80 (40.40)	6 (23.08)	74 (43.02)	
Three	54 (27.27)	7 (26.92)	47 (27.33)	
Person reside with				0.497
Guardian	35 (17.68)	4 (15.38)	31 (18.02)	
Parent	163 (82.32)	22 (84.62)	141 (81.98)	
Relationship with person residing with				0.303
Mother	51 (25.76)	3 (11.54)	48 (27.91)	
Father	8 (4.04)	0 (0.00)	8 (4.65)	
Both mother and father	103 (52.02)	19 (73.08)	84 (48.84)	
Auntie/uncle	17 (8.59)	2 (7.69)	15 (8.72)	
Grandparents	16 (8.08)	2 (7.69)	14 (8.14)	
Sister/step mother	3 (1.52)	0 (0.00)	3 (1.74)	
Occupation of guardian				0.072
Government employed	70 (35.35)	11 (42.31)	59 (34.30)	
Self‐employed	119 (60.10)	12 (46.15)	107 (62.21)	
Unemployed	9 (4.55)	3 (11.54)	6 (3.49)	
Religion				0.300
Christianity	172 (86.87)	24 (92.31)	148 (86.05)	
Islam	26 (13.13)	2 (7.69)	24 (13.95)	
Level of education of guardian				0.249
No formal education	24 (12.12)	4 (15.38)	20 (11.63)	
Formal education	136 (68.69)	19 (73.08)	117 (968.02)	
Informal education	35 (17.68)	2 (7.69)	33 (19.19)	
Others	3 (1.52)	1 (3.85)	2 (1.16)	
Ethnicity				0.328
Ashanti	35 (17.68)	7 (26.92)	28 (16.28)	
Fante	117 (59.09)	11 (42.31)	106 (61.63)	
Northerner	13 (6.57)	2 (7.69)	11 (6.40)	
Ga/Ewe/Akuapem/Krobo	11 (5.56)	2 (7.69)	9 (5.23)	
Mole Dagbani	18 (9.09)	3 (11.54)	15 (8.72)	
Foreigner	4 (2.02)	1 (3.85)	3 (1.74)	
Junior High School (JHS)				0.004
I. K. Islamic JHS (Amamoma)	36 (18.18)	2 (7.69)	34 (19.77)	
St. Anthony Anglican JHS (Akotokyir)	64 (32.32)	16 (61.54)	48 (27.91)	
Kwaprow M/A JHS (Kwaprow)	98 (49.49)	8 (30.77)	90 (52.33)	

*Note:* Data are expressed as the mean ± SD or *n* (%). All Chi‐square analyses are presented in row percentages. *Chi‐square *p*‐value; **Fisher's exact *p*‐value. Bold is used to highlight significant *p* values to enhance easy identification.

**Table 4:** Association between severe dysmenorrhea and the sociodemographic characteristics of female adolescent high school students.

Variable	Overall [*n* (%)]	Severe dysmenorrhea		*p*‐value**
		No [*n* (%)]	Yes [*n* (%)]	
Age (years) [mean ± SD = 14.45 ± 1.22]		14.28 ± 0.09	15.13 ± 0.21	0.0001†
Age category (years) [mean ± SD = 14.45 ± 1.22]				0.001
12–13 years	47 (23.74)	45 (95.74)	2 (4.26)	
14–16 years	139 (70.20)	108 (77.70)	31 (22.30)	
17–18 years	12 (6.06)	7 (58.33)	5 (41.67)	
Level/form				0.799
One	64 (32.32)	52 (81.25)	12 (18.75)	
Two	80 (40.40)	63 (78.75)	17 (21.25)	
Three	54 (27.27)	45 (83.33)	9 (16.67)	
Junior High School (JHS)				0.016
I. K. Islamic JHS (Amamoma)	36 (18.18)	32 (88.89)	4 (11.11)	
St. Anthony Anglican JHS (Akotokyir)	64 (32.32)	44 (68.75)	20 (31.25)	
Kwaprow M/A JHS (Kwaprow)	98 (49.49)	84 (85.71)	14 (14.29)	
Person reside with				0.120
Guardian	35 (17.68)	25 (71.43)	10 (28.57)	
Parent	163 (82.32)	135 (82.82)	28 (17.18)	
Relationship with person residing with				0.024
Mother	51 (25.76)	38 (74.51)	13 (25.49)	
Father	8 (4.04)	6 (75.00)	2 (25.00)	
Both mother and father	103 (52.02)	91 (88.35)	12 (11.65)	
Auntie/uncle	17 (8.59)	13 (76,47)	4 (23.53)	
Grandparents	16 (8.08)	9 (56.25)	7 (43.75)	
Sister/step mother	3 (1.52)	3 (100.00)	0 (0.00)	
Occupation of guardian				0.215
Government employed	70 (35.35)	61 (87.14)	9 (12.86)	
Self‐employed	119 (60.10)	92 (77.31)	27 (22.69)	
Unemployed	9 (4.55)	7 (77.78)	2 (22.22)	
Level of education of guardian				0.267
No formal education	24 (12.12)	17 (70.83)	7 (29.17)	
Formal education	136 (68.69)	110 (80.88)	26 (19.12)	
Informal education	35 (17.68)	31 (88.57)	4 (11.43)	
Others	3 (1.52)	2 (66.67)	1 (33.33)	
Religion				0.283
Christianity	172 (86.87)	141 (81.98)	31 (18.02)	
Islam	26 (13.13)	19 (73.08)	7 (26.92)	
Ethnicity				0.904
Ashanti	35 (17.68)	28 (80.00)	7 (20.00)	
Fante	117 (59.09)	96 (82.05)	21 (17.95)	
Northerner	13 (6.57)	10 (76.92)	3 (23.08)	
Ga/Ewe/Akuapem/Krobo	11 (5.56)	8 (72.73)	3 (27.27)	
Mole Dagbani	18 (9.09)	15 (83.33)	3 (16.67)	
Foreigner	4 (2.02)	3 (75.00)	1 (25.00)	

*Note:* Data are expressed as the mean ± SD or *n* (%). All Chi‐square analyses are presented in column percentages. **Fisher's exact *p*‐value. ^†^ *T*‐test *p*‐value. Bold is used to highlight significant *p* values to enhance easy identification.

Adolescents with irregular menstrual cycles had significantly higher prevalence of dysmenorrhea compared to those with regular cycles (*p* = 0.01) (Table 5). Severe dysmenorrhea was significantly associated with the frequency of menstrual cramps (*χ* ^2^ = 15.59, *p *= 0.002) and the duration of pain (*χ* ^2^ = 11.35, *p* = 0.001) (Table 6).

**Table 5:** Association between dysmenorrhea and the obstetric and gynaecological characteristics of adolescent high school females.

Variable	Overall [*n* (%)]	Dysmenorrhea		*p*‐value**
		No [*n* (%)]	Yes [*n* (%)]	
Age of menarche (years) [mean ± SD = 12.47 ± 1.04]		12.77 ± 0.99	12.43 ± 1.04	0.059†
Age or menarche categories				0.226
9–11 years	23 (11.68)	2 (8.70)	21 (91.30)	
12–13 years	149 (75.63)	18 (12.08)	131 (87.92)	
14–17 years	25 (12.69)	6 (24.00)	19 (76.00)	
Adolescent has regular cycle				0.010
No	98 (49.49)	6 (6.12)	92 (93.88)	
Yes	17 (8.59)	3 (17.65)	14 (82.35)	
Not sure	83 (41.92)	17 (20.48)	66 (79.52)	
Number of days of regular cycle (*n* = 17)				0.065
20–25 days	5 (29.41)	1 (20.00)	4 (80.00)	
26–28 days	8 (47.06)	0 (0.00)	8 (100.00)	
29–30 days	4 (23.53)	2 (50.00)	2 (50.00)	
Duration of blood flow				0.057
2 days or less	7 (3.54)	3 (42.86)	4 (57.14)	
3–5 days	116 (58.59)	16 (13.79)	100 (86.21)	
Greater than 5 days	75 (37.88)	7 (9.33)	68 (90.67)	
Number of pads usually used				0.713
Two or less	96 (48.48)	11 (11.46)	85 (88.54)	
Three to four	88 (44.44)	13 (14.77)	75 (85.23)	
Five or more	14 (7.07)	2 (14.29)	12 (85.71)	
Adolescent female circumcised				0.425
No	192 (96.97)	26 (13.54)	166 (86.46)	
Yes	6 (3.03)	0 (0.00)	6 (100.00)	
Having history of abortion				0.654
No	195 (98.48)	26 (13.33)	169 (86.67)	
Yes	3 (1.52)	0 (0.00)	3 (100.00)	

*Note:* Data are expressed as the mean ± SD or *n* (%). All Chi‐square analyses are presented in column percentages. **Fisher's exact *p*‐value; ^†^ *T*‐test *p*‐value. Bold is used to highlight significant *p* values to enhance easy identification.

**Table 6:** Association between severe dysmenorrhea and the obstetric and gynaecological characteristics of adolescent high school females.

Variable	Overall [*n* (%)]	Severe dysmenorrhea		*p*‐value**
		No [*n* (%)]	Yes [*n* (%)]	
Age of menarche (years) [mean ± SD = 12.47 ± 1.04]		12.47 ± 0.08	12.50 ± 0.21	0.855†
Age or menarche categories				0.787
9–11 years	23 (11.68)	19 (82.61)	4 (17.39)	
12–13 years	149 (75.63)	121 (81.21)	28 (18.79)	
14–17 years	25 (12.69)	19 (76.00)	6 (24.00)	
Adolescent has regular cycle				0.183
No	98 (49.49)	84 (85.71)	14 (14.29)	
Yes	17 (8.59)	12 (70.59)	5 (29.41)	
Not sure	83 (41.92)	64 (77.11)	19 (22.89)	
Number of days of regular cycle (*n* = 17)				1.000
20–25 days	5 (29.41)	3 (60.00)	2 (40.00)	
26–28 days	8 (47.06)	6 (75.00)	2 (25.00)	
29–30 days	4 (23.53)	3 (75.00)	1 925.00)	
Duration of blood flow				0.400
2 days or less	7 (3.54)	6 (85.71)	1 (14.29)	
3–5 days	116 (58.59)	97 (83.62)	19 (16.38)	
Greater than 5 days	75 (37.88)	57 (76.00)	18 (24.00)	
Crumps frequency (*n* = 172)				0.002
Rarely (1 or 2 times yearly)	12 (6.98)	12 (100.00)	0 (0.00)	
Occasionally (few times yearly)	54 (31.40)	46 (85.19)	8 (14.81)	
Monthly (each menstrual cycle)	101 (58.72)	75 (74.26)	26 (25.74)	
Frequently (> once monthly)	5 (2.91)	1 (20.00)	4 (100.00)	
Number of pads usually used				0.886
Two or less	96 (48.48)	77 (80.21)	19 (19.79)	
Three to four	88 (44.44)	72 (81.82)	16 (18.18)	
Five or more	14 (7.07)	11 (78.57)	3 (21.43)	
Pain duration (*n* = 172)				0.001
2 or less days	127 (73.84)	107 (84.25)	20 (15.75)	
3 days and more	45 (26.16)	27 (60.00)	18 (40.00)	
Adolescent female circumcised				0.086
No	192 (96.97)	157 (81.770	35 (18.23)	
Yes	6 (3.03)	3 (50.00)	3 (50.00)	
Having history of abortion				1.000
No	195 (98.48)	157 (80.51)	38 (19.49)	
Yes	3 (1.52)	3 (100.00)	0 (0.00)	

*Note:* Data are expressed as the mean ± SD or n (%). All Chi‐square analyses are presented in column percentages. **Fisher's exact *p*‐value; ^†^ *T*‐test *p*‐value. Bold is used to highlight significant *p* values to enhance easy identification.

The presence of dysmenorrhea was self‐reported to restrict adolescents from performing their normal physical activities (44.4%, *n* = 88). It led to poor concentration at school and home (39.4%, *n* = 78), social withdrawal (41.9%, *n* = 83), heightened irritability (42.9%, *n* = 85), a decrease in academic performance (15.2%, *n* = 30), and absenteeism (11.6%, *n* = 23) (Table 7). Adolescents with dysmenorrhea were restricted from their normal physical activities (*χ* ^2^ = 13.13, *p* < 0.001) (Table 8). Severe dysmenorrhea was associated with a decrease in the academic performance of adolescents. Adolescents who reported dysmenorrhea engaged in few activities as coping mechanisms. Notably, over‐the‐counter medications (26.8%, *n* = 53), ignoring pain (28.3%, *n* = 56), and rest (46.5%, *n* = 92) were mostly practiced while only a few visit the hospital (6.4%). We found a significant association between severe dysmenorrhea and the use of over‐the‐counter medications (*χ* ^2^ = 5.64, *p* = 0.02) and ignoring pain (*χ* ^2^ = 5.30, *p* = 0.02) as coping mechanisms (Table 9).

**Table 7:** Coping mechanisms and impact of dysmenorrhea on adolescent high school females.

Variable	Overall [*n* (%)]	Dysmenorrhea		*p*‐value**
		No [*n* (%)]	Yes [*n* (%)]	
*Effect of dysmenorrhea on social and academic life*				
It restricts me from my normal physical activities				< 0.0001
No	110 (55.56)	23 (20.91)	87 (79.09)	
Yes	88 (44.44)	3 (3.41)	85 (96.59)	
I have poor concentration in both school and home				0.163*
No	120 (60.61)	19 (15.83)	101 (84.17)	
Yes	78 (39.39)	7 (8.97)	71 (91.03)	
Social withdrawal				0.216*
No	115 (58.08)	18 (15.65)	97 (84.35)	
Yes	83 (41.92)	8 (9.64)	75 (90.36)	
Unnecessary irritation				0.621*
No	113 (57.07)	16 (14.16)	97 (85.84)	
Yes	85 (42.93)	10 (11.76)	75 (88.24)	
Decreased academic performance				0.558
No	168 (84.85)	21 (12.50)	147 (87.50)	
Yes	30 (15.15)	5 (16.67)	25 (83.33)	
I absent myself from school				0.049
No	175 (88.38)	26 (14.86)	149 (85.14)	
Yes	23 (11.62)	0 (0.00)	23 (100.00)	
Other effects				1.000
No	193 (97.47)	26 (13.47)	167 (86.53)	
Yes	5 (2.53)	0 (0.00)	5 (100.00)	
*Dysmenorrhoea coping mechanisms among high school females*				
Use of over‐the‐counter medications				0.234
No	145 (73.23)	22 (15.17)	123 (84.83)	
Yes	53 (26.77)	4 (7.55)	49 (92.45)	
Mostly ignore the pain				0.527*
No	142 (71.72)	20 (14.08)	122 (85.92)	
Yes	56 (28.28)	6 (10.71)	50 (89.29)	
I just rest				0.380*
No	106 (53.54)	16 (15.09)	90 (84.91)	
Yes	92 (46.46)	10 (10.87)	82 (89.13)	
Practice heat therapy				0.137
No	180 (90.91)	26 (14.44)	154 (85.56)	
Yes	18 (9.09)	0 (0.00)	18 (100.00)	
Consult a physician				0.365
No	187 (94.44)	26 (13.90)	161 (86.10)	
Yes	11 (5.56)	0 (0.00)	11 (100.00)	
Engage in physical activities				0.013*
No	174 (87.88)	19 (10.92)	155 (89.08)	
Yes	24 (12.12)	7 (29.17)	17 (70.83)	
Herbal remedies/alternative therapies				0.336
No	189 (95.45)	24 (12.70)	165 (87.30)	
Yes	9 (4.55)	2 (22.22)	7 (77.78)	
Other mechanisms				0.129
No	193 (97.47)	24 (12.44)	169 (87.56)	
Yes	5 (2.23)	2 (40.00)	3 (60.00)	
Pain medications used (*n* = 53)				0.693
Menstak	7 (13.21)	1 (14.29)	6 (85.71)	
Paracetamol	34 (64.15)	2 (5.88)	32 (94.12)	
Aspirin/Hyoscine/Ibuprofen	7 (13.21)	1 (14.29)	6 (85.71)	
Others (Gebedol/Efpac/Lydia)	5 (9.43)	0 (0.00)	5 (100.00)	
Effectiveness of pain medication (*n* = 53)				0.935
Not effective	5 (9.43)	0 (0.00)	5 (100.00)	
Poorly effective	9 (16.98)	0 (0.00)	9 (100.00)	
Mildly effective	15 (28.30)	2 (13.33)	13 (86.67)	
Moderately effective	10 (18.87)	1 (10.00)	9 (90.00)	
Highly effective	14 (26.42)	1 (7.14)	13 (92.86)	

*Note:* Data are expressed as the mean ± SD or *n* (%). All Chi‐square analyses are presented in column percentages. *Chi‐square *p*‐value; **Fisher's exact *p*‐value. Bold is used to highlight significant *p* values to enhance easy identification.

**Table 8:** Logistic regression of dysmenorrhea against significant sociodemographic characteristics and obstetric and gynaecological factors.

Variable	OR	95% CI	*p*‐value	AOR	95% CI	*p*‐value
Junior High School (JHS)						
St. Anthony Anglican JHS (Akotokyir)	Ref	Ref	Ref	Ref	Ref	Ref
I. K. Islamic JHS (Amamoma)	5.67	1.22–26.28	0.027	3.82	0.72–20.28	0.115
Kwaprow M/A JHS (Kwaprow)	3.75	1.50–9.39	0.005	1.49	0.34–6.54	0.597
Age of menarche (years)	0.73	0.49–1.09	0.120	0.51	0.28–0.91	0.023
Adolescent has regular cycle						
Not sure	Ref	Ref	Ref	Ref	Ref	Ref
No	3.95	1.48–10.55	0.006	1.74	0.36–8.33	0.489
Yes	1.20	0.31–4.67	0.790	0.36	0.06–2.08	0.254
It restricts me from my normal physical activities						
No	Ref	Ref	Ref	Ref	Ref	Ref
Yes	7.49	2.17–25.88	0.001	11.97	2.61–54.93	0.001
I absent myself from school						
No	Ref	Ref	Ref	Ref	Ref	Ref
Yes	Predicts success perfectly			Predicts success perfectly		
Engage in physical activities						
Yes	Ref	Ref	Ref	Ref	Ref	Ref
No	3.36	1.23–9.14	0.018	6.29	1.70–23.34	0.006

*Note:* Bold is used to highlight significant *p* values to enhance easy identification.

Abbreviations: AOR, adjusted odds ratio while controlling for all significant factors in order of appearance; CI, confidence interval; OR, odds ratio.

**Table 9:** Logistic regression of severe dysmenorrhea against significant sociodemographic characteristics and obstetric and gynaecological factors.

Variable	OR ± SE	95% CI	*p*‐value	AOR	95% CI	*p*‐value
Junior High School (JHS)						
St. Anthony Anglican JHS (Akotokyir)	Ref	Ref	Ref	Ref	Ref	Ref
I. K. Islamic JHS (Amamoma)	0.28 ± 0.16	0.09–0.88	0.03	0.11 ± 0.09	0.02–0.52	0.006
Kwaprow M/A JHS (Kwaprow)	0.37 ± 0.14	0.17–0.80	0.011	0.41 ± 0.22	0.14–1.15	0.091
Age (years)	1.75 ± 0.27	1.30–2.36	< 0.0001	1.46 ± 0.33	0.93–2.28	0.099
Age category (years)						
12–13 years	Ref	Ref	Ref	Ref	Ref	Ref
14–16 years	6.45 ± 4.85	1.48–28.13	0.01	4.01 ± 3.35	0.78–20.62	0.096
17–18 years	16.07 ± 14.95	2.60–99.48	0.003	2.92 ± 3.67	0.25–34.31	0.393
Relationship with person residing with						
Both mother and father	Ref	Ref	Ref	Ref	Ref	Ref
Mother	2.59 ± 1.15	1.09–6.20	0.032	3.54 ± 2.07	1.13–11.14	0.030
Father	2.53 ± 2.21	0.46–13.97	0.288	4.29 ± 4.86	0.46–39.56	0.199
Auntie/uncle	2.33 ± 1.51	0.65–8.32	0.192	2.36 ± 2.16	0.39–14.24	0.350
Grandparents	5.90 ± 3.48	1.86–18.75	0.003	8.31 ± 7.10	1.56–44.33	0.013
Crumps frequency (*n* = 172)						
Occasionally (few times yearly)	Ref	Ref	Ref	Ref	Ref	Ref
Monthly (each menstrual cycle)	1.99 ± 0.89	0.83–4.77	0.122	1.64 ± 0.87	0.58–4.66	0.354
Frequently (> once monthly)	23.00 ± 27.18	2.27–233.19	0.008	19.12 ± 29.88	0.89–409.07	0.059
Pain duration (*n* = 172)						
2 or less days	Ref	Ref	Ref	Ref	Ref	Ref
3 days and more	3.57 ± 1.39	1.66–7.65	0.001	3.92 ± 2.14	1.35–11.42	0.012
Decreased academic performance						
No	Ref	Ref	Ref	Ref	Ref	Ref
Yes	3.64 ± 1.56	1.57–8.45	0.003	3.70 ± 2.41	1.03–13.24	0.045
Use of over‐the‐counter medications						
No	Ref	Ref	Ref	Ref	Ref	Ref
Yes	2.42 ± 0.91	1.15–5.07	0.020	1.63 ± 0.83	0.60–4.43	0.337
Mostly ignore the pain						
Yes	Ref	Ref	Ref	Ref	Ref	Ref
No	3.09 ± 1.57	1.14–8.37	0.027	3.73 ± 2.40	1.05–13.20	0.042

*Note:* Bold is used to highlight significant *p* values to enhance easy identification.

Abbreviations: AOR, adjusted odds ratio while controlling for all significant factors in order of appearance; CI, confidence interval; OR, odds ratio.

Adolescents who experienced early menarche (9–11 years) had significantly lower odds of reporting severe dysmenorrhea compared to those with late menarche (14–17 years) [AOR = 0.51, 95% CI = 0.28–0.91, *p* = 0.02] (Table 9). Adolescents attending the Islamic JHS (AOR = 0.11 ± 0.09, 95% CI = 0.0–0.5, *p* = 0.006) and the Kwaprow JHS (OR = 0.37 ± 0.14, 95% CI = 0.2–0.8, *p* < 0.0001) had higher odds of reporting severe dysmenorrhea (Table 9).

Increasing age was significantly associated with increased odds of having severe dysmenorrhea. Adolescents residing with only their mother had approximately fourfold higher odds of reporting severe dysmenorrhea (AOR = 3.5 ± 2.1, 95% CI = 1.1–11.1, *p* = 0.03) and an eightfold‐fold increase in the odds of having dysmenorrhea if one resides with her grandparents (AOR = 8.3 ± 7.1, 95% CI = 1.6–44.3, *p* = 0.01) (Table 9).

## 4 Discussion

This study assessed the prevalence, effects, and coping mechanisms of dysmenorrhea among adolescent girls in JHSs in the Cape Coast Municipality. The prevalence of dysmenorrhea was 86.9% (*n* = 172). Approximately half of the participants (*n* = 98) had irregular menstrual cycles; dysmenorrhea was less likely to occur in adolescents who reported early menarche, and living with grandparents was associated with a higher likelihood of reporting dysmenorrhea. Dysmenorrhea significantly restricted adolescents from their normal physical activities; approximately 30% of the participants relied on over‐the‐counter medications for pain management, whereas only 6.4% consulted a physician.

We found that the prevalence of dysmenorrhea was 86.9%. This is in line with the established prevalence of dysmenorrhea (60%–93%) among the study population [6]. Compared with a similar study among adolescents in the Greater Accra region (prevalence of 68.1%) [3], we found an increased prevalence of dysmenorrhea. Nonetheless, our findings correlate well with studies by Ameade et al. (2018) in the northern region of Ghana [17] and other cross‐sectional studies in Lebanon [7] and Ireland [13].

Approximately half of the participants (*n* = 98) had irregular cycles, with only 8.6% (*n* = 17) confirming that they had regular menstrual cycles. This finding is comparable with the findings of studies conducted among adolescents in Ethiopia and among nursing students in Lebanon, which reported prevalence rates of 42.8% and 53.5%, respectively. This figure is high compared with the 25.7% of menstrual irregularities reported by Acheampong et al. [3] in Ghana and the 18.6% reported by Amu and Bamidele [19] in Nigeria. These variations may be attributed to differences in study populations, demographics and sample size. A significant proportion of participants (41.9%) reported uncertainty about their menstrual cycle regularity. This reflects limited menstrual health knowledge among adolescents and the need for heightened menstrual health education in schools. We found a significant relationship between the prevalence of dysmenorrhea and irregular menstrual cycles, which is in line with the findings of previous studies [3, 22]. Irregular menstrual cycles are often characterized by fluctuations in oestrogen and progesterone levels, which affect ovulation and prostaglandin production, leading to intense uterine contractions and pain. While our findings reinforce the association between irregular menstrual cycles and dysmenorrhea, the underlying mechanism remains complex and multifactorial.

Compared to those who had late menarche, adolescents who experienced early menarche were 50% less likely to report dysmenorrhea. In a study among adolescent girls in India, Patel and Barot [2] reported that women who reported early menarche were more likely to experience dysmenorrhea. However, there is some controversial evidence regarding the influence of early and late menarche on dysmenorrhea [23]. De Sanctis et al. [24], in a study among Italian adolescent girls, reported that menarchal age independently does not affect dysmenorrhea. The controversy may lie in the discrepancies in sample sizes and participants' sociodemographics. Given that dysmenorrhea is influenced by a combination of genetic [25], biological [26], environmental [8], and psychosocial factors [5, 27], isolating the effect of menarchal age is challenging. Age was not associated with the overall presence of dysmenorrhea in this study; however, it was associated with the severity of dysmenorrhea among affected adolescents. While dysmenorrhea occurred across all age groups, symptom severity increased with age.

Adolescents who stayed with their grandparents were 8.3 times more at risk of experiencing dysmenorrhea compared with those who stayed with both parents. This aligns with the findings of Acheampong et al. [3], who concluded that adolescents not living with their parents have significantly higher odds of self‐reported dysmenorrhea. Adolescents who do not reside with their parents face a greater risk of experiencing dysmenorrhea due to factors such as parental separation or loss and exposure to domestic conflicts or instability, all of which increase stress levels. Notably, we also found that the school environment influenced the prevalence of dysmenorrhea, with students at Islamic JHS and Kwaprow JHS demonstrating higher odds of reporting the condition. Owing to the already established connection between psychological stressors, environment and dysmenorrhea [5], it is plausible that differences in school community dynamics, menstrual stigma, and socioeconomic background may also contribute may influence dysmenorrhea severity. This merits further investigation with designs better suited to explore school‐level influences.

Dysmenorrhea significantly restricts the normal physical activities of adolescents, as supported by many studies [15, 27]. Nearly half (49.4%) of the participants who reported dysmenorrhea experienced physical activity limitations as a result of their condition. Abreu‐Sánchez et al. [9], in a study among Spanish nursing students, reported that certain daily activities, such as sitting, walking, and having a full bladder, caused or intensified menstrual pain among students with dysmenorrhea. Some studies have demonstrated that regular exercise can significantly reduce the severity and frequency of dysmenorrhea [28, 29]. Engaging in regular exercise induces hormonal changes in the uterine lining, which can alleviate the symptoms associated with dysmenorrhea [30]. Physical activity can increase the production of endorphins, which act as natural painkillers and improve blood flow to the pelvic region. In the current study, only 9.9% of the adolescents used physical activity as a coping mechanism for dysmenorrhea. This is comparable to the findings of Acheampong et al. [3] (14%). Given that physical activity can reduce pain intensity, it is imperative to promote public education on the benefits of exercise as an effective strategy for managing dysmenorrhea.

Dysmenorrhea is the primary cause of recurrent short‐term school absenteeism among menstruating adolescents [10, 11, 15]. This may affect their performance academically due to interference with concentration and performance during their period [12, 13]. We found that 13.4% of the adolescents with dysmenorrhea reported absenteeism, and 41.3% reported poor concentration at home and at school. These findings are consistent with those of previous studies reporting that 5%–14% of adolescents absent themselves from school due to dysmenorrhea [31]. School‐based interventions that provide menstrual health education and support systems to adolescents could significantly enhance their attendance and academic engagements.

Self‐medication, also known as over‐the‐counter medication, is widely practiced in both developed and developing countries, especially among menstruating women [32]. In this study, 29% of the adolescents ignored pain, and 28.4% relied on over‐the‐counter medications for pain management. Menstak, paracetamol, and ibuprofen are among the commonly used drugs. This corresponds with the findings of Acheampong et al. [3], who reported that 34.6% of adolescents relied heavily on self‐medication. Only 6.4% of the adolescents had consulted a physician for their menstrual pain. This aligns with many studies indicating that most adolescents with dysmenorrhea do not seek medical consultation [32]. The reluctance to consult a physician can be attributed to various factors, including the normalization of menstrual pain, lack of awareness about effective treatment options, and stigma surrounding menstrual health issues [33]. This gap in adolescent healthcare access and awareness necessitates initiatives that promote safe pain management practices and improve access to adolescent‐friendly reproductive health services. While over‐the‐counter pain relievers have proven to be effective in providing symptomatic relief for this population [1], increased access to medication also raises concerns about non‐responsible self‐medication [32]. Delays in seeking medical advice when needed, adverse drug–drug interactions, and improper dosages are among the few concerns that can arise from self‐medication [34]. Additionally, some diseases may have presentations similar to those of dysmenorrhea, which may lead to misdiagnosis and, consequently, improper treatment [35].

### 4.1 Limitations

We used a cross‐sectional design to collect self‐reported data on dysmenorrhea among adolescent girls. This makes our work prone to recall bias. Our sample size of 198 may also limit the generalizability of our findings. The majority of participants had not sought medical evaluation or consulted a healthcare professional, limiting our ability to definitively exclude secondary causes of dysmenorrhea. We did not adjust analyses of age at menarche for gynaecological age, which may influence dysmenorrhea severity.

## 5 Conclusion

Our study revealed that the incidence of dysmenorrhea is very high among adolescents in the Cape Coast municipality and adversely affects their social and academic lives. With nearly one‐third of adolescents relying on over‐the‐counter drugs and only 6.4% consulting physicians for menstrual pain, there are pervasive concerns of self‐medication. Additionally, this study revealed that psychosocial factors, such as living arrangements and the school environment, play critical roles in the prevalence and severity of dysmenorrhea. These findings highlight significant gaps in healthcare access and important impacts on education, emphasizing the need for better public health strategies.

## Author Contributions

Richard K. D. Ephraim and Attoh Tetteh conceived and designed this study. Amina S. Abugri was responsible for data collection and analysis. Gabriel P. Kotam and Angele Comlan‐Cataria drafted the manuscript. Gabriel P. Kotam, Attoh Tetteh, Amina S. Abugri, Stephen Ocansey, and Richard K. D. Ephraim revised the manuscript. Stephen Ocansey and Richard K. D. Ephraim supervised the entire process. All authors read and approved the final version of the manuscript. Gabriel Pezahso Kotam had full access to all of the data in this study and take complete responsibility for the integrity of the data and the accuracy of the data analysis.

## Funding

The authors received no specific funding for this work.

## Ethics Statement

This study was approved by the Department of Physician Assistant Studies, College of Health and Allied Sciences, University of Cape Coast. The participants were assured of the voluntary nature of the study and their freedom to withdraw at any point during the study if they had any personal reasons to discontinue. Our study protocol conforms to the provisions of the Declaration of Helsinki in 1995 (as revised in Edinburgh 2000).

## Consent

Consent was obtained from the Head teachers and guardians of all students recruited for the study.

## Conflicts of Interest

The authors declare no conflicts of interest.

## Transparency Statement

The lead author, Gabriel P. Kotam, affirms that this manuscript is an honest, accurate, and transparent account of the study being reported; that no important aspects of the study have been omitted; and that any discrepancies from the study as planned (and, if relevant, registered) have been explained.

## Data Availability

Data available upon request from the authors.
